# Correlation of *Aggregatibacter actinomycetemcomitans* Detection with Clinical/Immunoinflammatory Profile of Localized Aggressive Periodontitis Using a 16S rRNA Microarray Method: A Cross-Sectional Study

**DOI:** 10.1371/journal.pone.0085066

**Published:** 2013-12-23

**Authors:** Patricia F. Gonçalves, Vanja Klepac-Ceraj, Hong Huang, Bruce J. Paster, Ikramuddin Aukhil, Shannon M. Wallet, Luciana M. Shaddox

**Affiliations:** 1 Department of Dentistry, Federal University of Jequitinhonha and Mucuri Valleys, Diamantina, Minas Gerais, Brazil; 2 Department of Periodontology, University of Florida College of Dentistry, Gainesville, Florida, United States of America; 3 Department of Microbial Ecology and Pathogenesis, The Fortsyth Institute, Cambridge, Massachusetts, United States of America; 4 Department of Biological Sciences, Wellesley College, Wellesley, Massachusetts, United States of America; 5 Department of Oral Medicine, Infection and Immunity, Harvard School of Dental Medicine, Boston, Massachusetts, United States of America; Columbia University, United States of America

## Abstract

**Objective:**

The objective of this study was to determine whether the detection of *Aggregatibacter actinomycetemcomitans* (Aa) correlates with the clinical and immunoinflammatory profile of Localized Aggressive Periodontitis (LAP), as determined by by 16S rRNA gene-based microarray.

**Subjects and Methods:**

Subgingival plaque samples from the deepest diseased site of 30 LAP patients [PD ≥ 5 mm, BoP and bone loss] were analyzed by 16S rRNA gene-based microarrays. Gingival crevicular fluid (GCF) samples were analyzed for 14 cyto/chemokines. Peripheral blood was obtained and stimulated in vitro with *P.gingivalis* and *E.coli* to evaluate inflammatory response profiles. Plasma lipopolysaccharide (LPS) levels were also measured.

**Results:**

*Aa* was detected in 56% of LAP patients and was shown to be an indicator for different bacterial community structures (p<0.01). Elevated levels of pro-inflammatory cyto/chemokines were detected in LPS-stimulated blood samples in both *Aa*-detected and *Aa*-non-detected groups (p>0.05). Clinical parameters and serum LPS levels were similar between groups. However, *Aa*-non-detected GCF contained higher concentration of IL-8 than *Aa*-detected sites (p<0.05). TNFα and IL1β were elevated upon *E.coli* LPS stimulation of peripheral blood cells derived from patients with *Aa*-detected sites.

**Conclusions:**

Our findings demonstrate that the detection of *Aa* in LAP affected sites, did not correlate with clinical severity of the disease at the time of sampling in this cross-sectional study, although it did associate with lower local levels of IL-8, a different subgingival bacterial profile and elevated LPS-induced levels of TNFα and IL1β.

## Introduction

*Aggregatibacter actinomycetemcomitans* (Aa) is a Gram-negative bacterium that colonizes the oral cavity of one third or more of the population aged up to 18 years [1]. It has been highly implicated as the causative agent of aggressive periodontitis (AgP), a group of less frequent, often severe, rapidly progressive forms of periodontitis, with a more localized presentation of the disease (LAP) [2-4]. LAP is also characterized by an early age of onset, tendency for familial involvement, and bone loss affecting specifically molar and incisor teeth, [5]. Importantly, recent papers have pointed out the importance *Aa*, a particularly highly leukotoxic clone (JP2), in the initiation of attachment loss in young individuals [4,6,7].

Our group has recently demonstrated a hyper-inflammatory trait in response to bacterial endotoxin in an LAP cohort of African-Americans, which results in the over-expression of pro-inflammatory cyto/chemokines [8]. We also observed an exacerbated local inflammatory response, along with high systemic levels of bacterial lipopolysaccharides (LPS) [9]. Additionally, we have reported *Aa* to be highly associated with LAP in this cohort [10]. However, we could not detect *Aa* in every patient with LAP in our cohort, which is also consistent to previously reported literature [11]. Little is known regarding the impact of *Aa*’s presence on LAP disease presentation (clinical parameters and bacterial colonization) or the accompanying inflammatory response (regulation and local presentation). 

Thus, the objective of this study was to evaluate our LAP cohort in relation to the presence of *Aa* and determine whether the detection of this species in LAP affected sites correlate with clinical presentation of disease, local cyto/chemokines, plasma LPS levels and the level of hyper-responsiveness of these individuals.

## Materials and Methods

### Ethics statement

This study was conducted in full conformity with the principles set forth in The Belmont Report: Ethical Principles and Guidelines for the Protection of Human Subjects of Research, as drafted by the US National Commission for the Protection of Human Subjects of Biomedical and Behavioral Research (April 18, 1979) and codified in 45 CFR Part 46 and/or the ICH E6; 62 Federal Regulations 25691 (1997). This study was approved by the University of Florida Institutional Review Board.

### Subject demographics

**Figure 1:**
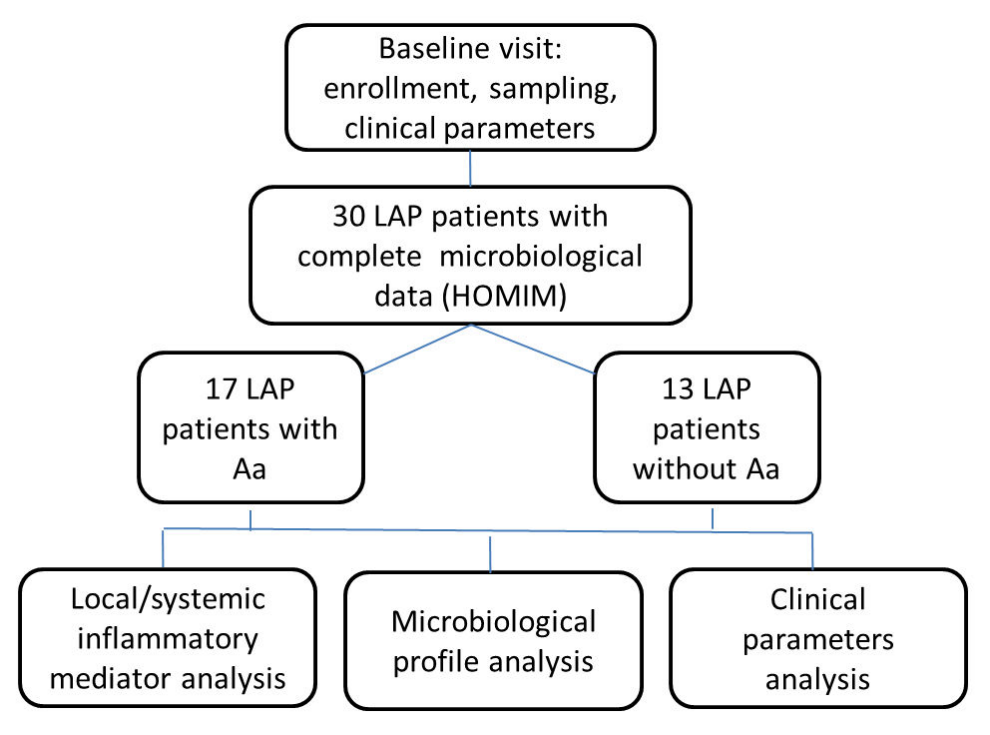
Schematic flowchart of study design.

Subjects were selected from a cohort recruited from Leon County Health Department, Tallahassee, Florida, from February 2007 to August 2010. This study is part of a larger clinical trial (Clinical trial registration: *#NCT01330719* at www.clinicaltrials.gov). A comprehensive microbiological profile as well as local and LPS-induced inflammatory response from initial subjects enrolled in this study has been reported in our previous publications [8-10]. Both historical data and newly generated data were part of the present analysis. Specifically, all subjects enrolled in the clinical trial that had their baseline microbiological analysis completed were included in the present report[10], and new data on local and systemic inflammatory markers for some of these patients were run to fulfill the present investigation (see flowchart, Figure 1). Eight of the participants had siblings within the cohort, who happen to be part of the random pool of participants on which microbiological analysis had been completed. All subjects and parents were informed about the study protocol and signed an informed consent previously approved by the University of Florida Institutional Review Board. Inclusion criteria: subjects aged 5-21 years old, African-American, diagnosed with localized aggressive periodontitis, defined by presence of at least 2 sites, on first molar and/or incisor, with pocket depth >4mm with bleeding on probing, presence of at least 2mm clinical attachment loss and radiographic bone loss [5,12]. Exclusion criteria: subjects diagnosed with any systemic diseases or conditions that could influence the progression and/or clinical characteristics of periodontal disease (e.g., diabetes or blood disorders); taken antibiotics within the last 3 months or any medications that could influence the characteristics of the disease (e.g., phenytoin, cyclosporine); have received periodontal treatment within the previous 6 months; smokers; and pregnant/lactating women. 

### Clinical measurements

The following periodontal clinical parameters were taken by one calibrated examiner at the baseline visit (prior to treatment): Pocket Depth (PD), Bleeding on probing (BoP); Gingival margin position (GM), Clinical attachment level (CAL); Plaque Index (PI). All measurements were performed using a UNC-15 periodontal probe at six sites per tooth and recorded using a computer software program (Florida Probe, Gainesville, FL, USA). Periapical and interproximal radiographs were taken on all patients at the baseline visit for diagnosis purposes only.

### Gingival Crevicular Fluid (GCF) sampling

Following clinical examination, and on the same day, samples were collected from the most severe diseased site (PD ≥ 5mm with BoP and CAL≥2 mm and radiographic bone loss). Sites for GCF collection were isolated with cotton rolls, gently air-dried and supragingival plaque removed. A collection strip (PerioPaper GCF collection strips, Oraflow Inc, Plainview, NY) was inserted in the sites 1-2 mm into the sulcus for ~10 sec, as described previously [9]. Volume of GCF was measured using aPeriotron^®^8000, (Oraflow, Inc.). GCF samples were frozen at -70°C until analysis of soluble mediators was performed. 

### Collection of bacterial sub-gingival biofilm

Subgingival plaque samples were collected from the same diseased site where GCF collection was made, as described above, also prior to treatment. The area of collection was isolated with cotton rolls and supragingival plaque was carefully removed. A sterile endodontic paper point was used for the collection, placed in an eppendorf tube and stored at -70°C until processed. 

### Microbial microarray analysis

DNA was isolated from plaque samples as previously described [10]. After purification, DNA concentration and quality was determined using the Nanodrop (ND-1000 Spectrophotometer, Nanodrop Technologies INC, Wilmington, DE, USA). Samples were analyzed at the Forsyth Institute using the Human Oral Microbe Identification Microarrays (HOMIM) as previously described [10,13]. Briefly, 16S rRNA genes were PCR amplified from DNA extracts using 16S rRNA universal forward and reverse primers and labeled via incorporation of Cy3-dCTP in a second nested PCR. HOMIM uses16S rRNA-based, reverse-capture oligonucleotide probes (typically 18 to 20 bases), which are printed on aldehyde-coated glass slides and probed with labeled PCR products described above which are hybridized in duplicate. The microarray slides are scanned using an Axon 4000B scanner and crude data is extracted using GenePix Pro software (Molecular Devices, Sunnyvale, CA). Microbial profiles were generated from image files of scanned arrays using a HOMIM online analysis tool (http://bioinformatics.forsyth.org/homim/). Detection of a particular taxon was determined by presence of a fluorescent spot for that unique probe. A mean intensity for each taxon was calculated from hybridization spots of the same probe and signals were normalized by comparing individual signal intensities to the average of signals from universal probes and calculated as described previously [13]. Any original signal that was less than two times the background value was reset to 1 and was assigned to the signal level 0. Signals greater than 1 were categorized into scores from 1 to 5, corresponding to ranked signal levels. Presence of *Aa* was determined if a site showed value of 1 or above. Absence of *Aa* was determined if a site showed a value of ‘0’

### In vitro LPS stimulation

Peripheral blood was collected in heparinized tubes, diluted 1:4 in RPMI1640 (Invitrogen, Carlsbad, CA, USA) and stimulated with 1 μg/mL of ultrapure *E.coli* (Ec) LPS or *P.gingivalis* (Pg) LPS (InvivoGen, San Diego, CA, USA), or left untreated, for 24hrs [8]. After which the resulting supernatants were collected and stored at -70°C until analysis of soluble mediators was performed. 

### Analysis of soluble mediators

Multiplex assays (Milliplex^®^ 14-plex cytochemokine detection kits, Millipore, St. Charles, MO, USA) were used to detect and quantify 14 cyto/chemokines (Eotaxin, TNFα, IFNγ, IL1β, IL2, IL6, IL7, IL8, IL10, IL12p40, MCP-1, G-CSF, GM-CSF and MIP1α) in the crevicular fluid and LPS-stimulated culture supernatants according to the manufacture’s protocol. Data was acquired on the Luminex^®^200™ (Millipore, St. Charles, MO) and analyzed with Milliplex Software (Viagene Tech, Carlisle, MA, USA), a standard curve and 5-parameter logistics. Data is reported as pg/ml. GCF data is normalization to total protein content: normalized pg/ml = [pg/ml x protein content corrective ratio], where corrective ratio = [lowest protein concentration/protein concentration of sample of interest].

### Plasma LPS levels

Peripheral blood was collected in heparinized tubes, after which . plasma was separated from red blood cells by centrifugation (~300 x *g* for 15 min) and stored at -70°C. LPS levels were detected and quantified by using a chromogenic assay (Endpoint Chromogenic LAL Assay, Lonza, Basel, Switzerland). Endotoxin units/ml were calculated using a standard curve and best fit linear trend line, as previously described [9]. 

### Statistical analysis

Means were calculated and t-test was performed to compare patients presenting *Aa-detected* sites versus patients presenting *Aa-non-detected* sites for clinical and immunoinflammatory parameters. Mann-Whitney Rank Sum Test was applied when data was not normally distributed. ANOVA analyses were used for comparisons within groups for LPS stimulation using different ligands. This study was powered at >80% to detect a 10% difference between the two groups in cyto/chemokine values [values based on previous publication data [8,9]] and a PD difference in affected sites of at least 1 mm with standard deviation of 0.9 mm. SigmaPlot version 11.0 software (Systat Software Inc, Chicago, IL) was used for statistical analysis, considering a 5% significance level. We considered cyto/chemokines to be independent variables and thus, no statistical corrections were done here [14]. Canonical correspondence analysis (CCA), nonmetric multidimensional scaling (NMDS), and ANOVA tests were used on HOMIM hybridization scores to test for significant effects of environmental variables on microbial community structures within sites with or without the detection of *Aa*, using the Vegan package for the R statistical programming environment [15]. For generating CCA plots, *Aa* was excluded from the list of taxa. To account for the multiple hypotheses, we adjusted p-value using Benjamini/Hochberg correction [16]. 

## Results

### Clinical and microbiologic characterization

HOMIM resulted in 189 probes identifying at least 137 different bacterial taxa in diseased sites of LAP (for a more comprehensive microbial data description, including healthy controls, see [10]). Among these, two taxa of *Aa* were identified: *Aa*_ot531_AA84 and *Aa*_ot531_P02. Sites presenting any signal for *Aa* (value 1 or greater) were considered *Aa*-detected (n=17) and those that did not present any signal for *Aa* (value 0) were considered *Aa*-non-detected (n=13). HOMIM analysis’ mean relative signal intensity of *Aa* in the *Aa-detected*sites was 3.81±1.02, in a 0 to 5 signal scale. Interestingly, clinical parameters and demographics were similar between individuals with *Aa-detected* or *Aa-non-detected* sites, as determined by HOMIM (Table 1)*.*

**Table 1:** Clinical and demographic parameters for *Aa*+(positive) and *Aa*-(negative) groups.

	**Age**	**M/F**	**%PD>4mm**	**%BoP**	**%Plaque**	**Mean PD sites/all(mm)**	**Mean CAL(mm)**	**PD site (mm)**
***Aa*+**	13.19±3.94	8m/9f	12.76±7.81	19.41±10.05	43.64±25.88	5.02±0.71 /2.27±0.40	3.94±1.53	5.94±1.19
***Aa*-**	15.85±2.67	6m/7f	15.69±11.60	32.84±49.08	45.92±26.85	4.89±0.70/ 2.45±0.44	3.29±1.43	6.61±1.55
***P value***	0.371^*^	0.891^†^	0.416^*^	0.834^†^	0.986^†^	0.608^*^/0.258^*^	0.243^†^	0.220^†^

Values are given by Means ± Standard deviation. M=male; F=female; Mean PD sites= Mean pocket depth of sites with PD>4mm; Mean PD all= mean PD of all sites; CAL=clinical attachment level of affected sites; BoP=bleeding on probing; PD site = Pocket depth from site sampled for microbiological analysis. *t-test; ^†^Mann-Whitney Rank Sum Test.

**Figure 2:**
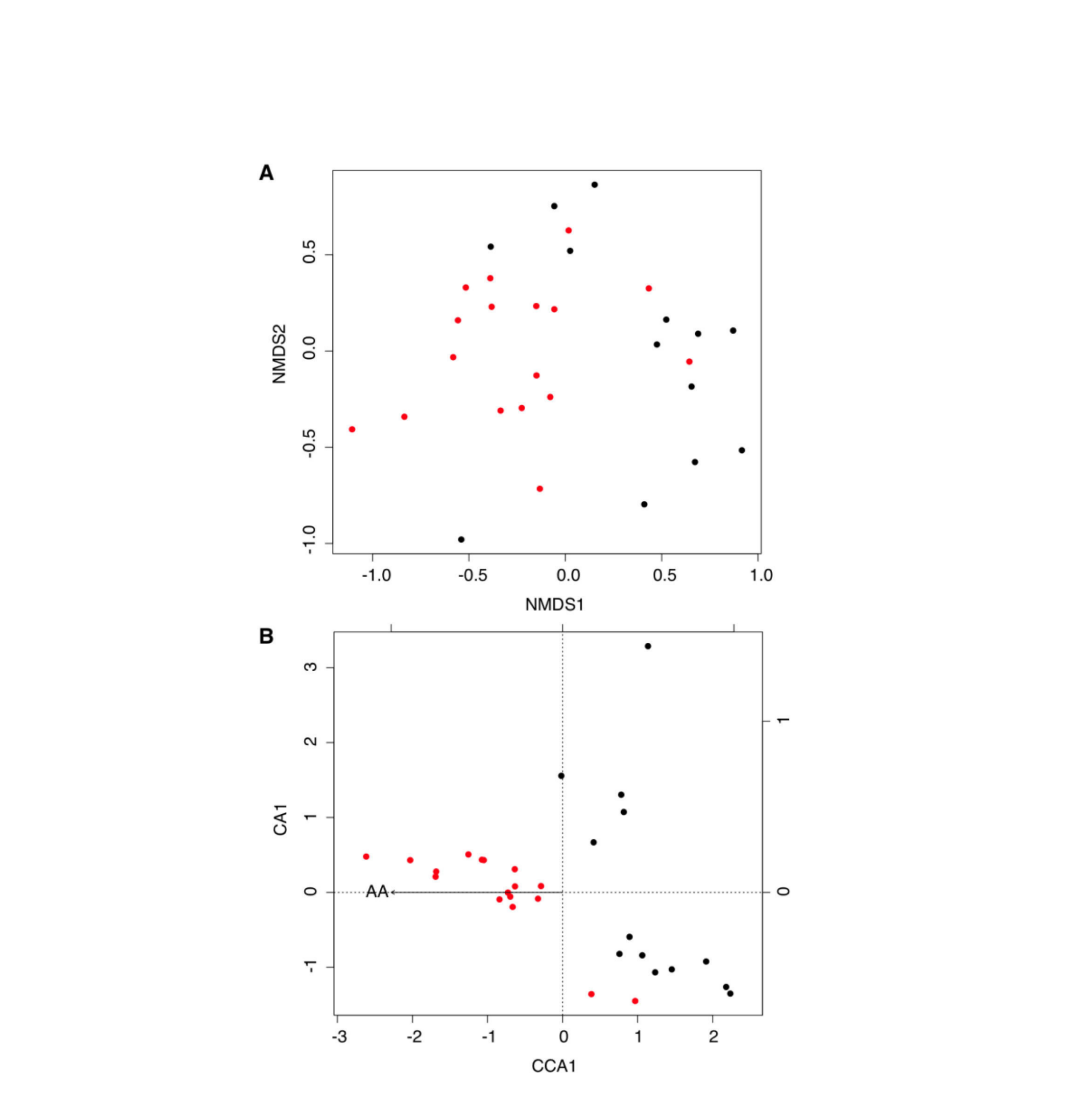
Effect of *Aa* and microbial community composition, as measured by HOMIM hybridization scores. The following variables were chosen as descriptors of the subgingival biofilm community and were calculated for each sample: pocket depth (PD), and presence of *Aa*. This last term was chosen to determine if *Aa* alone reflects shifts in community composition. A. Non-metric multidimensional scaling analysis (NMDS) and B. Canonical correspondence analysis (CCA) ordination diagrams of microbial communities. Red circles indicate samples where *Aa* was detected by HOMIM, and black circles indicate samples where there was no *Aa* detected by HOMIM analysis. *Aa* was critical for determining bacterial community structures (p<0.01).

In NMDS and CCA plots (Figure 2), samples with similar community composition cluster close together, while samples with different community compositions are farther apart on the plots; preserving the rank order of the original similarities [17]. Both NMDS and CCA analysis suggest that the detection of *Aa* was associated with specific bacterial community structures (of all variables tested, only the presence of *Aa* was statistically significant, permutation test under the reduced model with p<0.01). PD was not statistically significant relative to *Aa* detection (p=0.24) and therefore was not included in CCA. The left side of the CCA ordination plot is predominantly occupied by diseased sites containing *Aa*, whereas on the right side are predominantly diseased sites in which *Aa* was not detected. We observed that *Granulicatella elegans* (p=0.01) and *Prevotella loeschii* (p=0.02) were frequently found in samples co-existing when *Aa* was present. In contrast, *Porphyromonas gingivalis* (p=0.02) and *Parvimonas micra* (p=0.03) were more likely to be found in *Aa-non-detected* communities. These significances disappear when corrected for multiple comparisons. 

### Local inflammatory profile and systemic LPS levels

**Figure 3:**
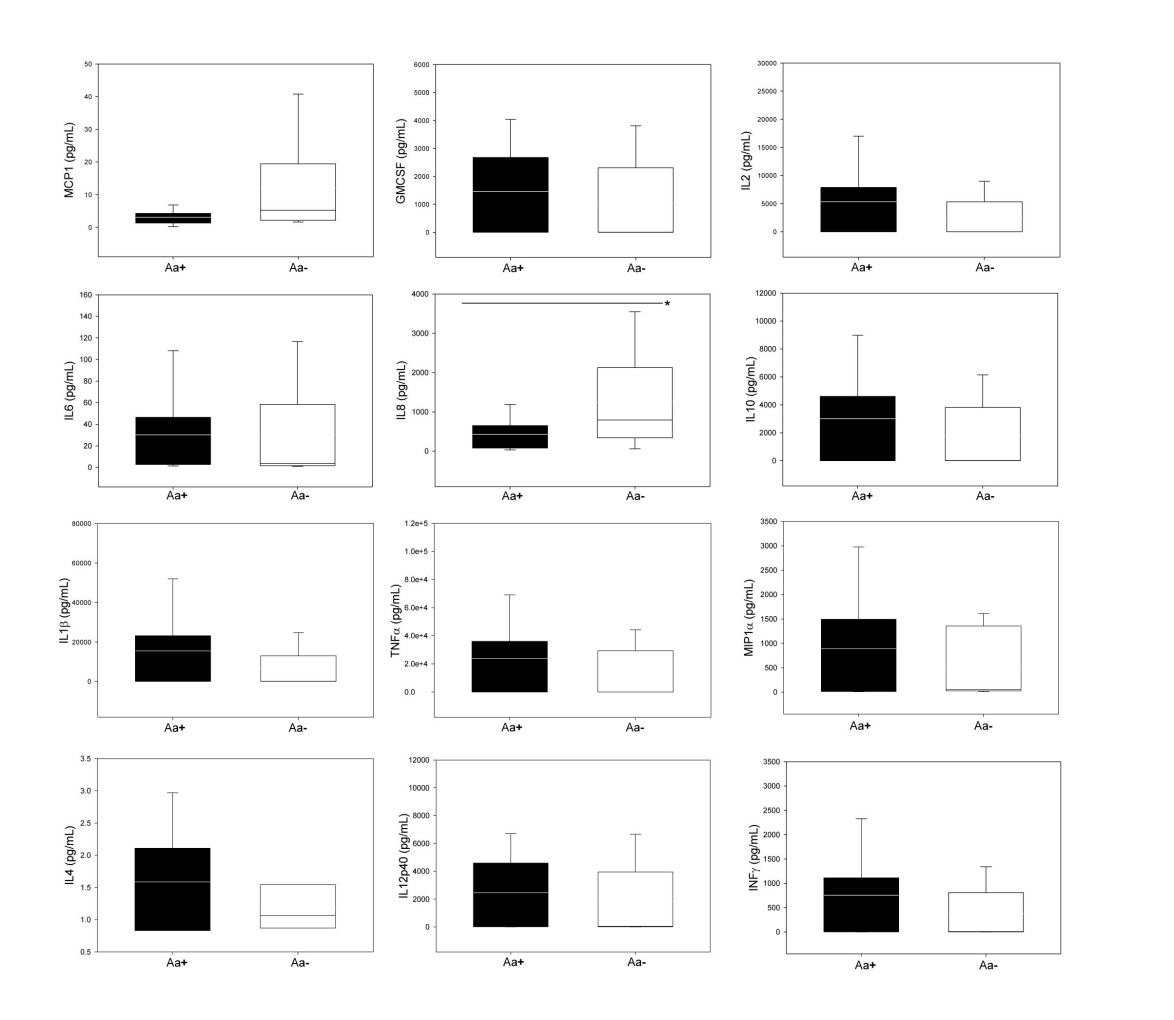
Inflammatory mediator profile in the GCF, in both groups. Cytochemokines values are reported as normalized pg/mL. Box-plots show the mean (horizontal line), inter-quartile range (box), 5^th^ -95^th^ percentiles (vertical lines). *Statistically significant (p=0.01), t-test.

As previously published, high levels of several inflammatory mediators were detected in the GCF of diseased sites of LAP participants [9]. Eotaxin, G-CSF and IL7 were not detected in high enough levels and thus were not included in the association analysis. In general, no differences in the GCF mediator profiles were detected between *Aa-detected* and *Aa-non-detected* sites (Figure 3). However, IL8 was found at higher concentrations in *Aa-non-detected* sites than those observed in *Aa-detected* sites (p=0.011). After controlling values for GCF volume at each diseased site of collection, the same trends were observed, and p-values slightly decreased, but did not reach statistical significance. Systemic LPS levels were similar in *Aa-non-detected* and *Aa-detected* groups (Mean values 0.61±0.15 and 0.68±0.36, respectively; p=0.773).

### LPS-induced hyper-responsive phenotype

**Figure 4:**
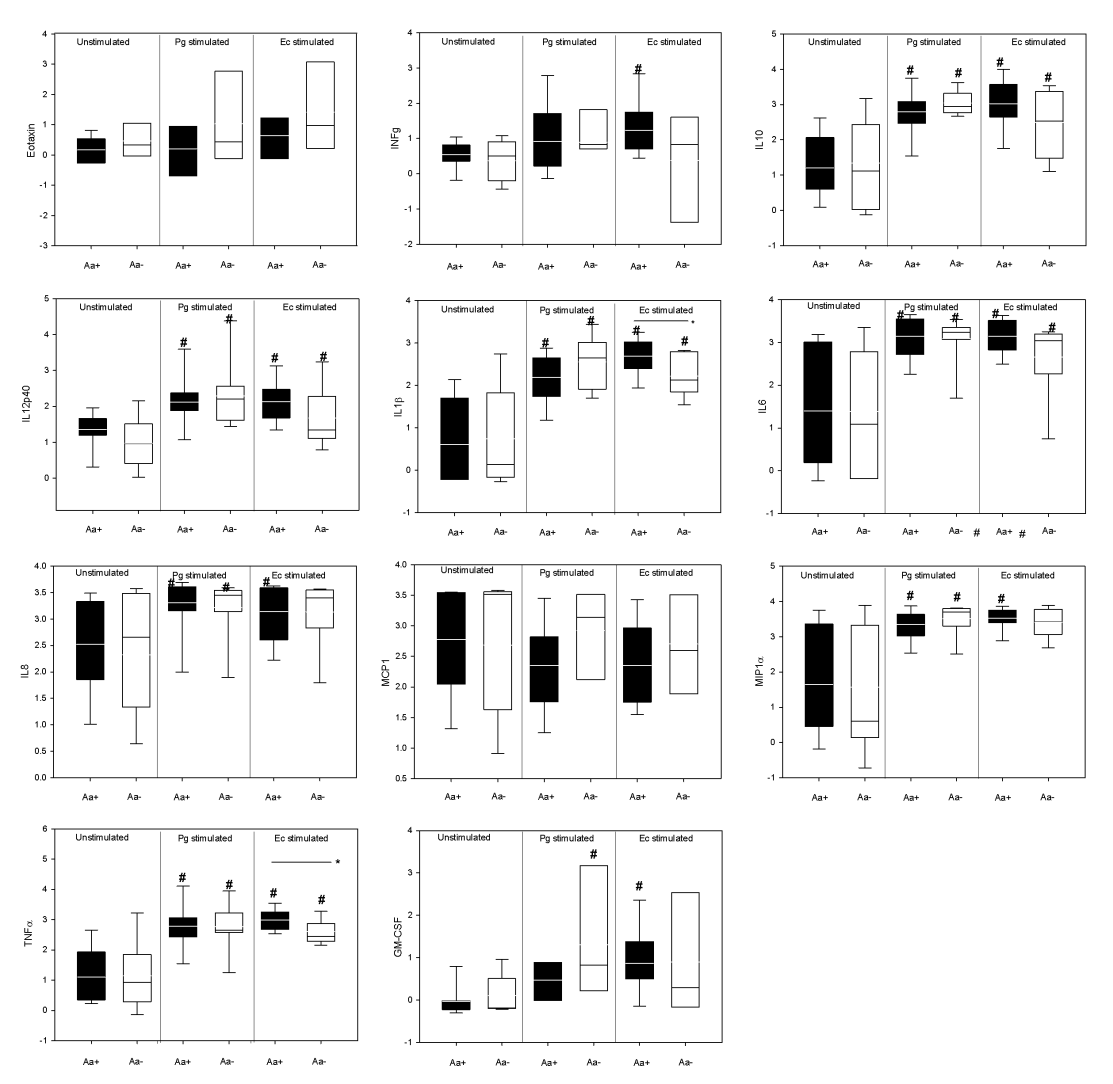
Systemic LPS-induced cyto/chemokine values in both groups. Values are reported as normalized log pg/mL. Box-plots show the mean (horizontal line), inter-quartile range (box), 5^th^ -95^th^ percentiles (vertical lines). *Indicates statistically significant differences between groups (Aa+ and Aa-) by t-test (p<0.05). **^*#*^**Indicates statistically significant differences within groups regarding TLR stimulation by One-way ANOVA (p<0.05).

The LPS responsiveness of individuals with *Aa-non-detected* and *Aa-detected* groups was compared. There was no significant differences between the levels of cyto/chemokines in unstimulated cultures of *Aa-detected* and *Aa-non-detected* individuals. Pg and Ec LPS-stimulation of whole blood cell preparation resulted in elevated levels of multiple pro-inflammatory cyto/chemokines when compared with unstimulated cultures (p<0.05). There were few significant differences in the levels of cyto/chemokines induced when comparing cohorts which had *Aa-detected* or *Aa-non-detected* sites. Specifically, Ec LPS stimulation elicited more robust levels of TNFα and IL1β from peripheral blood of individuals with *Aa-detected*sites than those with *Aa-non-detected* sites (p<0.05) (Figure 4). In addition, GM-CSF was more highly induced by Pg LPS-stimulation in individuals with *Aa-non-detected* sites, compared to those with *Aa-detected* sites, although the difference did not reach significance (Figure 4).

## Discussion

It has been previously suggested that an individual organism can play a strong role in community restructuring, and in turn impact the ecological processes of the system [18,19]. This change in community structure can also have an effect on local immune responses and thus the course of periodontal disease progression. Among periodontal pathogens, *Aa* has received particular attention and has been regarded as a key factor in the etiology of LAP. This view was based primarily on classic association studies linking *Aa* to disease and correlating treatment outcomes to its levels after therapy [20-25]. However, in a systematic review [11], it was shown that the presence or absence of *Aa* could not discriminate between subjects with aggressive periodontal disease from those with chronic periodontitis. 

The present study focused on the presence of *Aa* as a determinant factor in some aspects of disease presentation. We observed a lack of association between *Aa* detection, as measured by HOMIM, and severity of clinical signs of the disease (i.e. pocket depth, clinical attachment levels at time of sampling). This is not unexpected, considering that the criteria for diagnosis of LAP used in this study are very strict for clinical signs of disease, although it is impossible to determine the disease state of the sampled sites, even before treatment is rendered. However, if we were to include healthy individuals or even healthy sites (i.e. PD≤3mm and no bop), the presence of *Aa* could indeed be more correlated with the diseased clinical parameters [4,6,7,10,26]. It should be noted that the HOMIM assay has a sensitivity of about 0.1% and an overall limit of detection about 10^4^ cells (Colombo, 2009). Consequently, it is possible that *Aa* at levels below the level of detection could still be present. Nevertheless, *Aa-detected* sites presented very high signal levels for (mean relative signal intensity was 3.81), which makes the two populations very distinct for *Aa* detection using HOMIM. Interestingly, at this detection level, the presence or absence of *Aa* was associated with a difference in the composition of the microbial community as a whole. 

We also evaluated the correlation between the detection of *Aa* and local inflammatory mediators as a measure of active disease. Interestingly, a higher expression of IL-8 in GCF was detected in *Aa-non-detected* sites as compared to *Aa-detected* sites. IL-8 is involved in the recruitment of PMNs and is highly expressed in the junctional epithelium adjacent under the conditions of health whereby disease has been shown to modulate these levels both positively and negatively. Many organisms, including *P.gingivalis*, have virulence factors which affect IL8 stability within the GCF. Our data indicate that the non-detection of *Aa* results in a different microbial community than when it is detected at levels above 10^4^, thus it is possible that the absence/minimal presence of Aa, i.e. *Aa*-*non-detected* sites promotes an environment for an organism that possess these virulence factors. As demonstrated in our results, *P.gingivalis* may be frequently detected when *Aa* is not, where IL-8 levels were lower. Although *P.gingivalis* have been associated with periodontal inflammatory response, its potential to induce this response may shift according to the environment, specific virulent factors, and association with other species of the biofilm[27]. For instance, specific structures on *P.gingivalis* LPS may up-regulate or down-regulate levels of pro-inflammatory markers, including IL-8[28]. In addition, the presence of other bacterial species [29], as well as different peptides [30,31] may also reduce this organism’s pro-inflammatory abilities. On the other hand, it is possible that *Aa* has a direct effect on the suppression of IL8 from the junctional epithelium. However, the differences in IL8 expression in the GCF did not correlate with differences in clinical parameters. Both *Aa-detected* and *Aa-non-detected* cohorts had already advanced clinical signs of LAP, thus IL8 levels may be more indicative of active vs. inactive disease than severity of disease, which would also suggest that the presence and/or absence of *Aa* may also be a correlate of disease activity, although data presented here are insufficient to support this hypothesis. Longitudinal studies of the disease need to be conducted to asses this hypothesis.

Elevated local inflammation can sometimes result in the dissemination of bacteria and their products systemically. Interestingly, no differences in the levels of plasma LPS was observed between *Aa-detected* and *Aa-non-detected* cohorts. This was not unexpected considering that other than IL8, which does not directly contribute to epithelial ulceration, there were no significant differences in the levels of cyto/chemokines within the GCF of the *Aa-detected* and *Aa-non-detected* cohorts.

We have previously published that LAP individuals present with a hyper-inflammatory phenotype whereby they respond in a more robust manner to stimuli [8]. Here we demonstrate that overall whether an individual is colonized with *Aa* or not does not correlate with their level of LPS-induced responsive potential, with one exception. As a group, individuals with diseased sites that have *Aa detected*, respond more robustly to Ec LPS-stimulation as measured by IL1β and TNFα. *E.coli* LPS is mainly a TLR4 agonist, as is *Aa* LPS [32] [33] and this is why it was chosen for evaluation in the present study. Thus, one could hypothesize that in *Aa* carriers the potential for hyper-secretion of TNFα and IL1β could result in more robust tissue damage and disease progression. However, we did not observe elevated levels of TNFα nor IL1β in the GCF of *Aa detected* sites, nor was the snapshot of disease severity different in those individuals with *Aa-detected* or *Aa-non-detected* sites. The direct relationship between *Aa* presence, GCF markers and host inflammatory response to LPS cannot be resolved in this cross-sectional study but could be determined in future studies that test the likelihood that these cytokine elevations could be found prior to disease or during disease progression at the local site. 

In summary, the present study demonstrates that, in this population, similar clinical parameters of disease were observed regardless of whether *Aa* was detected or remained undetected by HOMIM in diseased sites. This study is cross-sectional and thus, it only represents a snapshot of the disease state at one point in time. However, our findings indicate potential differences in the microbial community profile associated (*G.elegans* and *P.loeschii*) or not associated (*P.gingivalis* and *P.micra*) with this species as well as effects on the local inflammation (IL8 in GCF). Interestingly, individuals with detectable levels of *Aa* also present with a TLR4 (*Ec* LPS) induced TNFα and IL1β hyper-responsiveness. Further longitudinal studies are necessary to evaluate whether these differences could impact disease progression and treatment responses.
